# Nonlinear optical properties of near-infrared region Ag_2_S quantum dots pumped by nanosecond laser pulses

**DOI:** 10.3762/bjnano.6.182

**Published:** 2015-08-24

**Authors:** Li-wei Liu, Si-yi Hu, Yin-ping Dou, Tian-hang Liu, Jing-quan Lin, Yue Wang

**Affiliations:** 1 School of Science, Changchun University of Science and Technology, Changchun, Jilin, 130022, China; 2 International Joint Research Center for Nanophotonics and Biophotonics, Changchun, Jilin, 130022, China

**Keywords:** nonlinear optics, quantum dots, silver sulfide (Ag2S), strong absorption

## Abstract

This study investigates near-infrared region Ag_2_S quantum dots (QDs) and their nonlinear optical response under 532 nm nanosecond laser pulses. Our experimental result shows that nonlinear transmission is reduced from 0.084 to 0.04. The observed narrowing behavior of the output pulse width shows superior optical limiting. We discuss the physical mechanisms responsible for the nonlinear optical response of the QDs. The average size of the nanocrystals was 5.5 nm. Our results suggest the possibility of using these Ag_2_S QDs for photoelectric, biosensor, optical ranging, and self-adaptive technologies.

## Introduction

Quantum dots (QDs) are semiconductor nanostructures with a size from 3 to 10 nm [1]. Through tailoring their size and composition, the emission wavelength of the quantum dots can be tuned from 650 to 1250 nm. In the last ten years, the study and development of QDs has rapidly progressed, and also influenced other research areas, such as nonlinear optics, plasmonics and biosensors. The special optical properties of QDs include good resistance to photo-bleaching, large absorption cross section, long fluorescence lifetimes, high quantum yields, and luminescence emission with large Stokes shift [2]. The development of synthetic nanoparticles has facilitated research on the nonlinear behavior of QDs and shown the great potential of QDs for different applications [3–4]. The synthesis of QDs with nonlinear behavior has been accomplished in aqueous solution over the past two decades [5–6]. Recently, studies have reported on nonlinear optical properties of QDs. Given their potential for various applications, including nonlinear reflectivity, efforts have recently been devoted to identifying QDs with near-infrared region (NIR) emission, such as PbSe, PbS, and CuInS_2_ [7–8]. These are non-cadmium-based QDs, and the use of NIR light solves the autofluorescence problem through the reduction of the fluorescence background [9–11]. Previous research has demonstrated that Ag_2_S QDs may be good candidates for use as NIR emitters [12–19]. Ag_2_S QDs have a band gap of 1.1 eV, the appropriate narrow band gap for NIR emission, and a relatively large absorption coefficient, which may enhance the emission intensity. Therefore, Ag_2_S QDs provide a powerful route for improving the optical properties of semiconductor nanocrystals [20–22]. Ag_2_S QDs have a wide range of applications in optical and other research fields. The development of their synthesis method may facilitate their application in optoelectronic devices for light absorption, photoconductor cells, IR detectors and so on [21,23–28].

Various optical properties of semiconductor or metallic nanoparticles with different sizes and shapes, such as nonlinear absorption and scattering, have been investigated both theoretically and experimentally [29–35]. However, there is a lack of research on the nonlinear properties of laser-pumped NIR QDs. In this study, we report experimental results of nonlinear optical behavior of Ag_2_S QDs pumped by 532 nm nanosecond laser pulses. The results of spectral, spatio-temporal and pump waveform measurements from laser pump energy effect are presented in this study and explained on the basis of the nonlinear optics theory of nanoparticles.

## Results and Discussion

**Figure 1:**
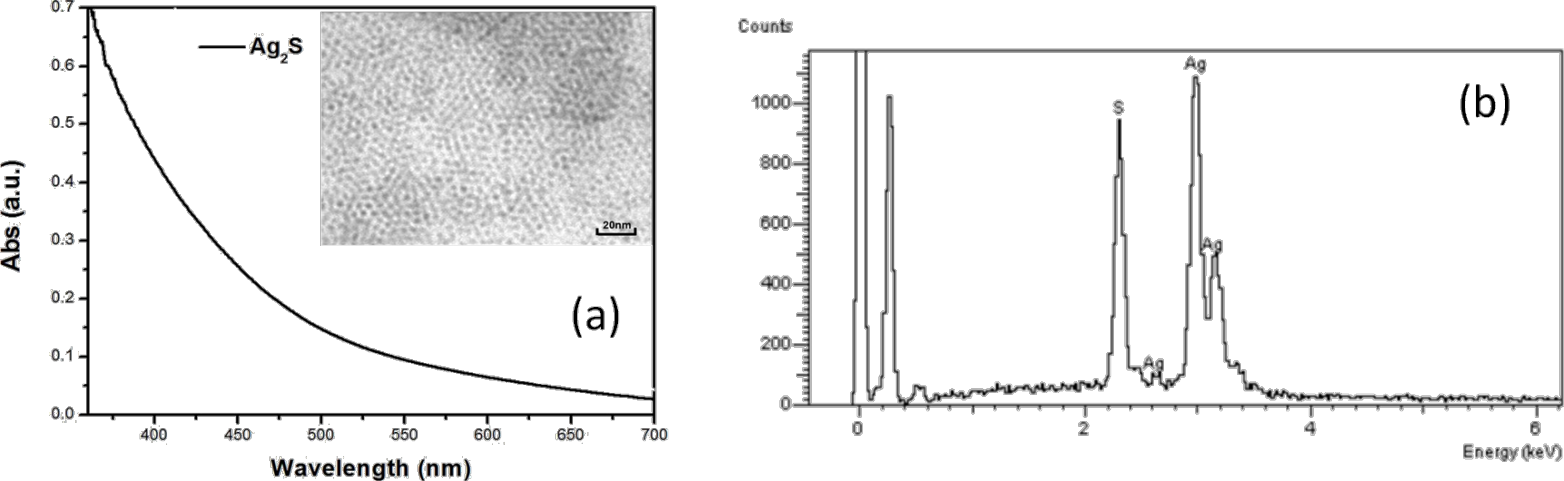
(a) Absorption spectra of Ag_2_S QDs in chloroform, the TEM image of Ag_2_S QDs is shown in the inset. (b) EDS spectra of Ag_2_S QDs.

Figure 1 shows the analysis of a sample of Ag_2_S nanocrystals dispersed in chloroform (CHCl_3_). The QDs were synthesized using the typical procedure published previously [20]. The sizes of the QDs were determined using a JEOL JEM-100cx transmission electron microscope (TEM) with an accelerating voltage of 80 kV. The TEM image of Ag_2_S is shown in Figure 1a. The particles are estimated to have an average diameter of 5–6 nm.

Figure 1a shows the absorption spectra of Ag_2_S QDs, measured by a scanning spectrophotometer (UV-3101PC from Shimadzu). Figure 1b shows the EDS spectra of Ag_2_S QDs clearly indicating Ag and S as constituents. The two sharp peaks at 0 and 0.15 keV have no relation to the Ag_2_S quantum dots, and are caused by a measurement error.

**Figure 2:**
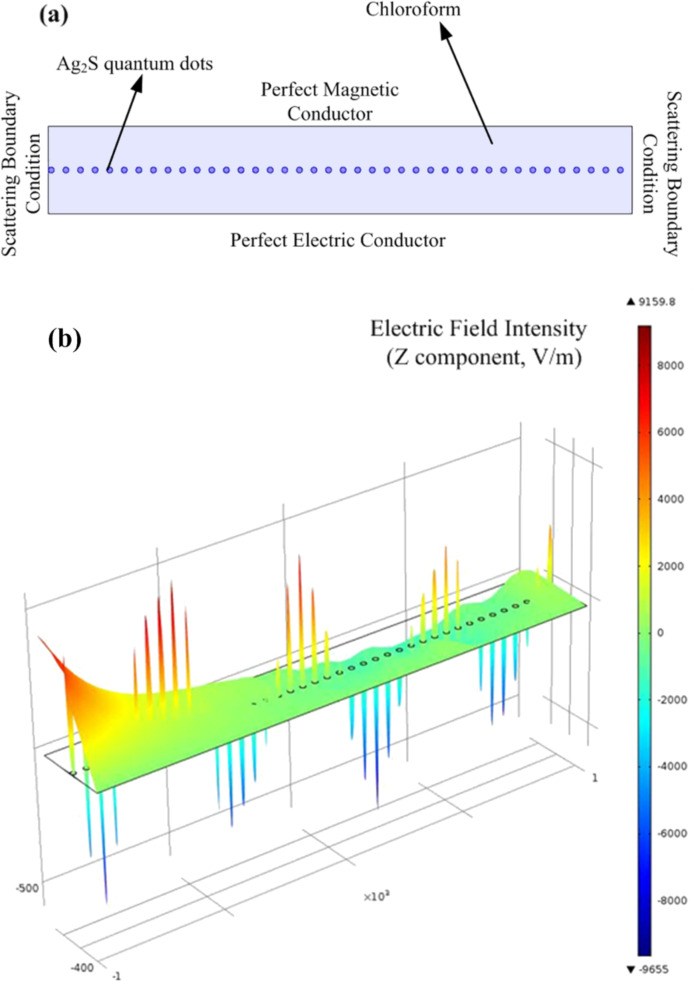
Simulation model (a) and result (b).

COMSOL Multiphysics 4.2a was used to simulate nonlinear optical characteristics for Ag_2_S QDs. Full-wave 2D analysis with Gaussian laser beam profile was employed. In order to reduce the needed computational resources, the model size was defined as 300 × 2000 nm, and the circles represent the quantum dots, as shown in Figure 2a. The following equations were used to describe the electric field (z-component):

[1]
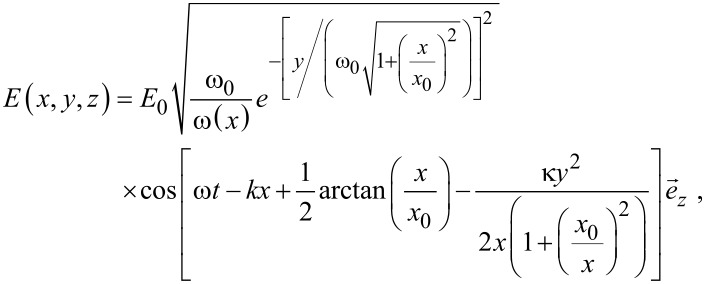


[2]
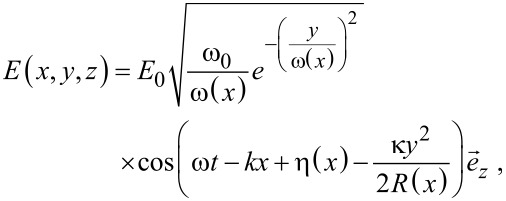


where ω_0_ is the minimum waist, ω is the angular frequency, *y* is the in-plane transverse coordinate, and *k* is the wave number. The simulation parameters are shown in Table 1.

**Table 1:** Simulation parameters.

name	value	description

ω_0_	2 [μm]	minimum waist
λ_0_	532 [nm]	laser wavelength
E*_0_*	30 [kV/m]	peak electric field
*x*_0_	π·ω_0_^2^/λ_0_	Rayleigh range
*k*_0_	2π/λ_0_	propagation constant
ω_0_	*k*_0_·*c*	Angular frequency
*dt*	8.9 [ns]	pulse width

The results of simulations are presented in Figure 2b, the dots in the rectangle represent the Ag_2_S QDs. One can see that the Ag_2_S QDs can show nonlinear optical phenomena under 532 nm laser irradiation. The intensity of the electric field changes drastically when the laser passes through the quantum dots. Previous research attributed the nonlinear optical behavior of nanoparticles to the reverse saturable absorption, when the nanoparticle were pumped by nanosecond laser pulses at a wavelength of 532 nm [36]. When the nanoparticles were pumped by femtosecond or picosecond laser pulses at wavelengths of 800 or 1064 nm, the nanoparticles exhibited self-focusing effects or positive nonlinear absorption, which reduced the nonlinear optical signal of nanoparticles [36–40].

**Figure 3:**
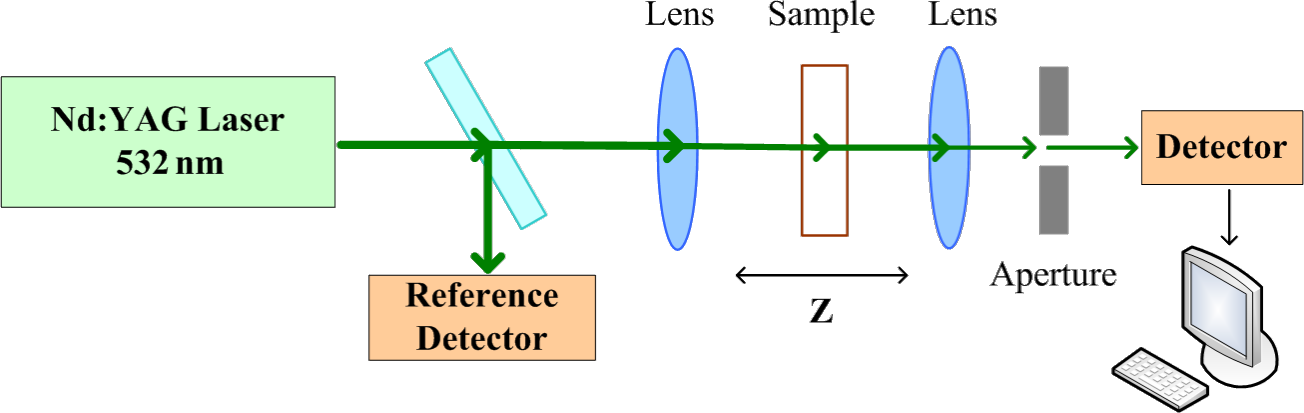
Experimental setup.

Figure 3 shows the experimental setup. The pump source is a 532 nm Nd:YAG laser with tunable pulse width [41]. The repetition rate was 10 Hz. Input pump pulses were focused with an *f* = 5 cm lens onto the center of a quartz cuvette with the sample solution. The optical path length of the focused pump beam passing through the sample medium was 2 cm, and the minimum waist of the laser is 3 ± 1 μm, the Rayleigh range of the laser is 53.12 μm.

**Figure 4:**
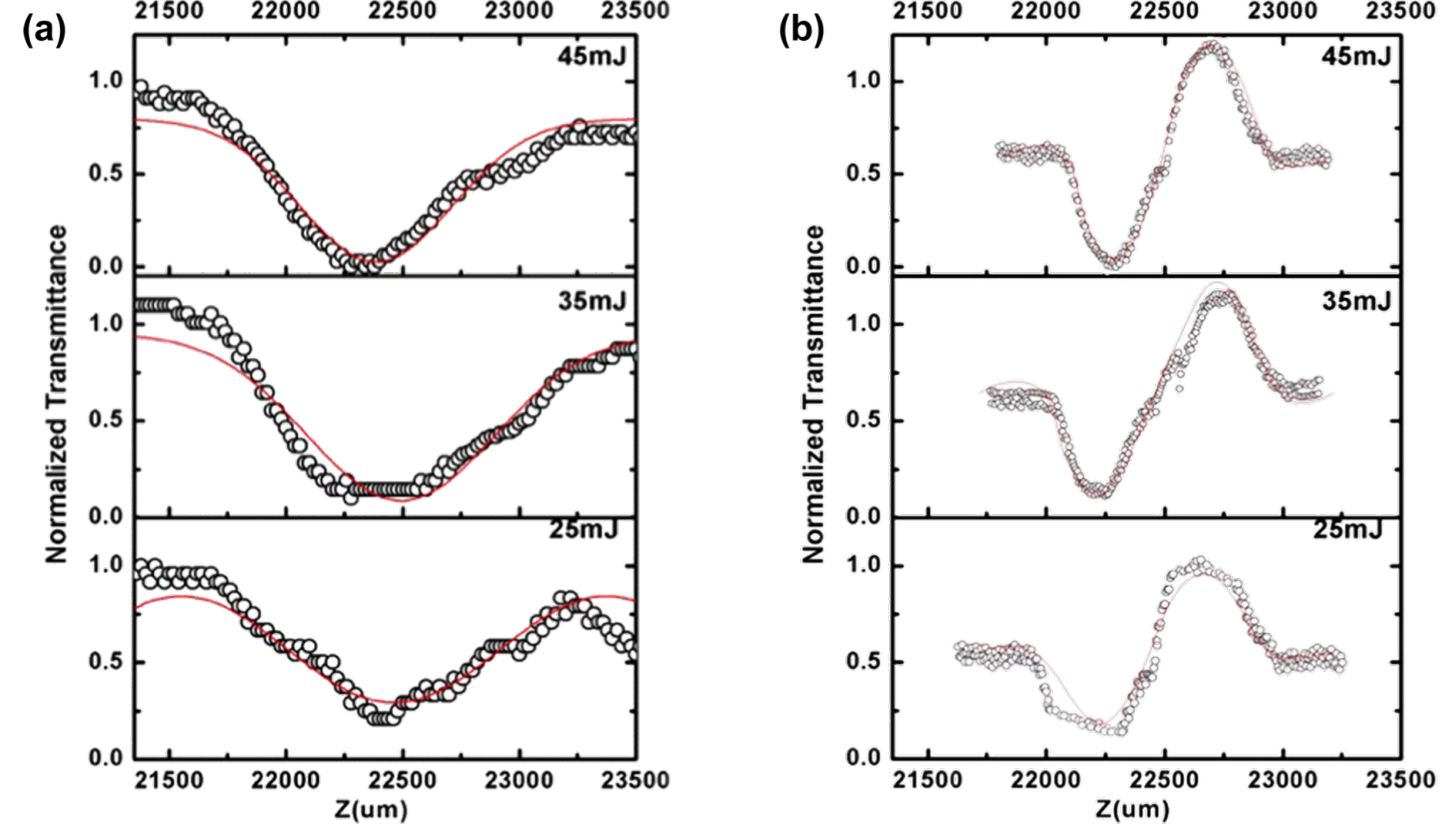
(a) Open-aperture *Z*-scan graph of Ag_2_S QDs; (b) closed-aperture *Z*-scan graph of Ag_2_S QDs.

Briefly, a *Z*-scan is one of the commonly utilized methods to measure nonlinear behavior. In this study, we used “open-aperture” and “closed-aperture” experiments according to Ganeev et al. [40]. Moreover, to check for the presence of nonlinear scattering, we used a photodiode placed at a fixed angle of about 40°. Insignificant nonlinear scattering of the laser beam was observed. Figure 4 shows the recorded *Z*-scan graphs of the Ag_2_S QDs. The laser pump energy ranged from 25 to 45 mJ.

We can see that all the open-aperture Z-scan exhibit a transmission maximum at low laser energy, indicating a saturable absorption behavior. However, there is a dip in the center of the transmission maximum with the laser energy increasing [42]. There is also a transmission minimum at higher laser energy, indicating a reverse saturable absorption behavior [22]. The curves of the closed-aperture *Z* -scans show different shapes compared to the open-aperture *Z*-scans, which is because of the combination of nonlinear effects in the system [22]. When the laser interacts with the QDs, free-carrier absorption, nonlinear scattering, and saturable absorption will occur, which all contribute to nonlinear behavior.

In semiconductor QDs the absorption coefficient and cross section were found to be the major properties that are largely affected by the structure of the semiconductor. Table 2 and Table 3 list the fitted nonlinear optical parameter of Ag_2_S QDs for open-aperture Z-scan and closed-aperture Z-scan. The data in the table have an estimated error of 15%, which is caused by the energy fluctuations of the laser beam and air turbulences.

**Table 2:** Fitted nonlinear optical parameter of Ag_2_S QDs for open-aperture *Z*-scans.^a^

laser energy (mJ)	linear absorption coefficient α (cm^−1^)	estimated size (nm)	absorption coefficient β_2_ (×10^−3^ cm/GW)	cross section σ_2_ (×10^4^ GW)

25	0.32	5.2	1.889	3.899
35	0.23	5.2	0.686	1.416
45	0.16	5.2	0.376	0.775

^a^All values listed have an estimated error of 15%.

**Table 3:** Fitted nonlinear optical parameter of Ag_2_S QDs for closed-aperture *Z*-scans.^a^

laser energy (mJ)	linear absorption coefficient α (cm^−1^)	estimated size (nm)	absorption coefficient β_2_ (×10^−3^ cm/GW)	cross section σ_2_ (×10^4^ GW)

25	0.32	5.2	1.193	2.462
35	0.23	5.2	0.305	0.629
45	0.16	5.2	0.095	0.195

^a^All values listed have an estimated error of 15%.

We measured the pulse energy values of the input and the transmitted laser beams to get a larger detecting area compared to the beam cross section. In our case, the dynamic transmission of the sample is determined by [41]:

[3]
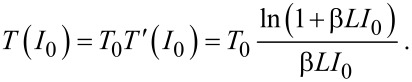


Here, *T*_0_ is the linear transmission, T′ is the nonlinear transmission, *I*_0_ is the input pulse energy, and the absorption coefficient *β* was obtained through Equation 4:

[4]
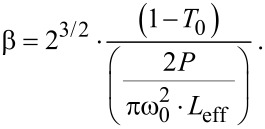


Here, *T*_0_ is the normalized peak height, *P* is the pulse peak power, ω_0_ is the beam waist radius, *L*_eff_ is the effective length in medium. The cross section σ was obtained by Equation 5:

[5]
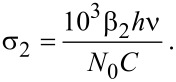


**Figure 5:**
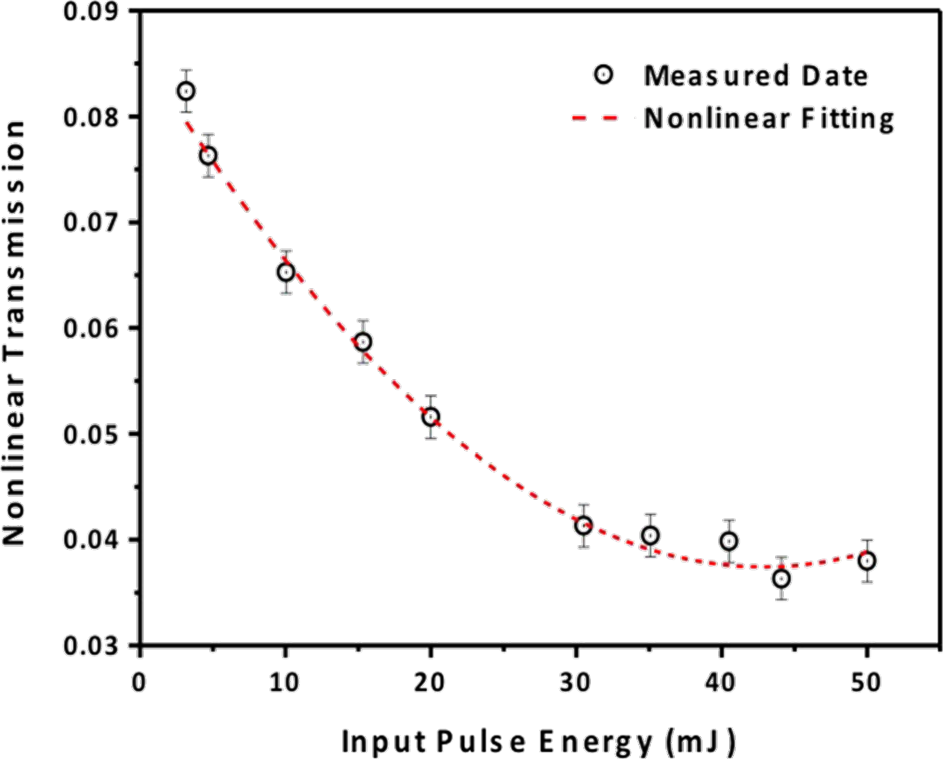
Measured nonlinear transmission versus the input pulse energy for Ag_2_S QDs. The dashed line represents the best fitting curve based on Equation 3 with β = constant.

**Figure 6:**
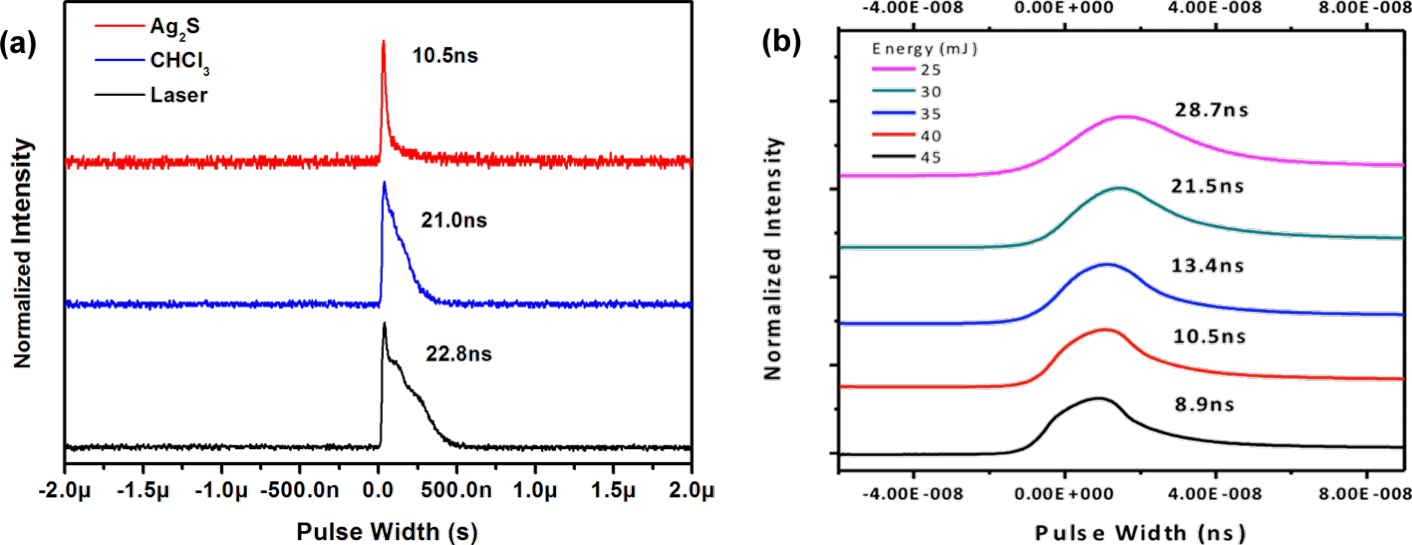
(a) Normalized waveforms of laser pulse and transmission pulse at the same pump energy level; (b) normalized waveforms of transmission pulse of Ag_2_S quantum dots showing width curve at different pump energy.

Here, *C* is concentration, *h*ν is the energy of incident light, *N*_0_ is a molecular number of quantum dots solutions. We set *L* as the constant optical path in the sample. As shown in Figure 5 we can see that the measured nonlinear transmission value is reduced from 0.084 to 0.04 when the input pulse energy increased. Figure 6a shows the waveforms of pulse and transmission pulse measured at the same pump energy level (40 mJ) by a two-channel digital oscilloscope of 200 MHz bandwidth (TDS 2024B).

It can be seen that the laser pulse width is 22.8 ns for a pump energy of 40 mJ. The transmission pulse is 10.5 ns for Ag_2_S QDs, and 21.08 ns for CHCl_3_. The transmission pulse of nanostructure particles is much shorter than that of the laser pulse. Figure 6b shows the transmission pulses at a different pump energy level. The waveforms became narrower as the laser pump energy increased. In a two-level system, such as this, when the laser pulse width is much larger than the particle relaxation time, multiple absorption will occur in the QDs. For Ag_2_S QDs, a large Ag protrusion is formed in the nanogap when the laser pump energy increased. Both electronic and ionic currents contribute to the absorption of the Ag_2_S QDs, which is affected by the pulse width waveforms. It could also be explained by an effect of nonlinear optical absorption that depends othe pump energy. This may reflect the dynamic behavior of gain or feedback mechanisms in the nonlinear absorption behavior of nanostructure particles. To exclude the possibility of a nonlinear behavior of nanocrystalline materials, we conducted the experiment for Ag_2_S QDs absorption measurement.

**Figure 7:**
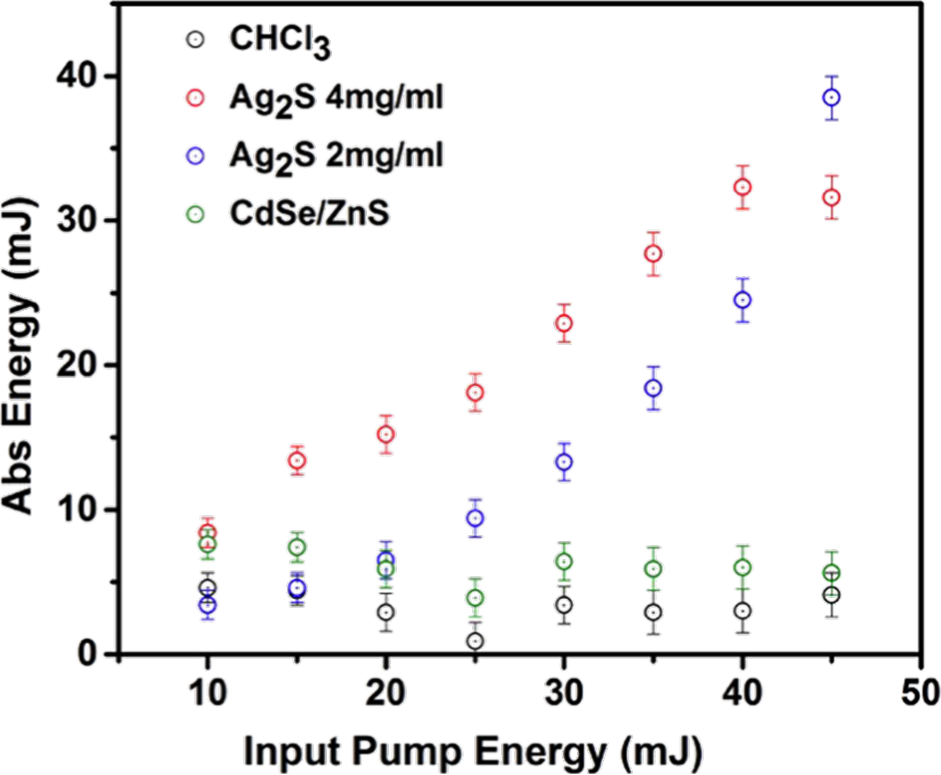
Absorption intensity and input pump energy spectra of Ag_2_S.

Figure 7 shows the output energy as a function of the input pump energy at different concentration values. The intensity of the absorption is defined as the ratio of the output energy to the input pump energy. It is shown that at the different pump levels, the Ag_2_S sample with a higher concentration (4.0 mg/mL) produced a smaller output energy than the Ag_2_S sample with a lower concentration (2.0 mg/mL). CHCl_3_ produced the lowest absorption intensity. Ag_2_S exhibited stronger absorption compared with CHCl_3_ as the pump energy increased. This can be explained by the fact that there are many impurities and electron–hole pairs in QDs. Also, when the laser interacts with QDs the dielectric constant of the medium surrounding the QDs will be changed. Ag_2_S has a narrow band gap, which leads to the strong absorption characteristics of Ag_2_S and which makes Ag_2_S particularly suitable for use in optical detectors and solar cells.

## Conclusion

In summary, we have demonstrated, using NIR Ag_2_S QDs pumped by 532 nm nanosecond pulses, that the nonlinear optical characteristics can be efficiently generated in QDs. The simulation results show the nonlinear characteristics of Ag_2_S QDs. Nonlinear transmission measurement revealed that Ag_2_S QDs have a superior nonlinear optical performance because of their strong absorption characteristics, and can compress pump pulse waveform. The strong absorption characteristics of Ag_2_S QDs may lead to more uses for nonlinear applications. This makes them promising candidates for nonlinear optical studies and the potential future applications of nanoparticles. We also note that in future research we may explore further the specific physical mechanisms and dynamics of nanoparticles with laser technology.

## References

[1] Prasad P N (2004). Nanophotonics.

[2] Liu L, Hu R, Law W-C, Roy I, Zhu J, Ye L, Hu S, Zhang X, Yong K-T (2013). Analyst.

[3] Kim K S, Hur W, Park S-J, Hong S W, Choi J E, Goh E J, Yoon S K, Hahn S K (2010). ACS Nano.

[4] Vivas M G, Cury J F, Schiavon M A, Mendonca C R (2013). J Phys Chem C.

[5] Bothun G D, Rabideau A E, Stoner M A (2009). J Phys Chem B.

[6] Rivera Gil P, Parak W J (2008). ACS Nano.

[7] Wehrenberg B L, Wang C, Guyot-Sionnest P (2002). J Phys Chem B.

[8] Bakueva L, Gorelikov I, Musikhin S, Zhao X S, Sargent E H, Kumacheva E (2004). Adv Mater.

[9] Kim S, Lim Y T, Soltesz E G, De Grand A M, Lee J, Nakayama A, Parker J A, Mihaljevic T, Laurence R G, Dor D M (2004). Nat Biotechnol.

[10] Balet L P, Ivanov S A, Piryatinski A, Achermann M, Klimov V I (2004). Nano Lett.

[11] Blackman B, Battaglia D, Peng X (2008). Chem Mater.

[12] Liu Z, Tabakman S, Sherlock S, Li X, Chen Z, Jiang K, Fan S, Dai H (2010). Nano Res.

[13] Smith A M, Mancini M C, Nie S (2009). Nat Nanotechnol.

[14] Masuo S, Naiki H, Machida S, Itaya A (2009). Appl Phys Lett.

[15] Yeh Y-C, Yuan C-T, Kang C-C, Chou P-T, Tang J (2008). Appl Phys Lett.

[16] Yuan C T, Chou W C, Chuu D S, Chen Y N, Lin C A, Chang W H (2008). Appl Phys Lett.

[17] Allen P M, Bawendi M G (2008). J Am Chem Soc.

[18] Welsher K, Liu Z, Sherlock S P, Robinson J T, Chen Z, Daranciang D, Dai H (2009). Nat Nanotechnol.

[19] Welsher K, Sherlock S P, Dai H J (2011). Proc Natl Acad Sci U S A.

[20] Du Y, Xu B, Fu T, Cai M, Li F, Zhang Y, Wang Q (2010). J Am Chem Soc.

[21] Peng P, Sadtler B, Alivisatos A P, Saykally R J (2010). J Phys Chem C.

[22] Liaros N, Aloukos P, Kolokithas-Ntoukas A, Bakandritsos A, Szabo T, Zboril R, Couris S (2013). J Phys Chem C.

[23] Terabe K, Hasegawa T, Nakayama T, Aono M (2005). Nature.

[24] Gao F, Lu Q, Zhao D (2003). Nano Lett.

[25] Huxter V M, Mirkovic T, Nair P S, Scholes G D (2008). Adv Mater.

[26] Lou W, Wang X, Chen M, Liu W, Hao J (2008). Nanotechnology.

[27] Xiang J, Cao H, Wu Q, Zhang S, Zhang X, Watt A A R (2008). J Phys Chem C.

[28] Hirsch M P (1998). Environ Toxicol Chem.

[29] Lee K-S, El-Sayed M A (2005). J Phys Chem B.

[30] Jain P K, Lee K S, El-Sayed I H, El-Sayed M A (2006). J Phys Chem B.

[31] Aslan K, Holley P, Davies L, Lakowicz J R, Geddes C D (2005). J Am Chem Soc.

[32] Evanoff D D, Chumanov G (2004). J Phys Chem B.

[33] Bogatyrev V A, Dykman L A, Khlebtsov B N, Khlebtsov N G (2004). Opt Spectrosc.

[34] Yguerabide J, Yguerabide E E (1998). Anal Biochem.

[35] He G S, Zhu J, Yong K-T, Baev A, Cai H-X, Hu R, Cui Y, Zhang X-H, Prasad P N (2010). J Phys Chem C.

[36] Fan G, Qu S, Wang Q, Zhao C, Zhang L, Li Z (2011). J Appl Phys.

[37] Zeng H, Yang Y, Jiang X, Chen G, Qiu J, Gan F (2005). J Cryst Growth.

[38] Ganeev R A, Boltaev G S, Tugushev R I, Usmanov T (2010). Appl Phys B.

[39] Ganeev R A, Ryasnyansky A I, Stepanov A L, Usmanov T (2004). Opt Quantum Electron.

[40] Ganeev R A, Suzuki M, Baba M, Ichihara M, Kuroda H (2008). J Appl Phys.

[41] He G S, Oh H S, Prasad P N (2011). Opt Lett.

[42] Neo M S, Venkatram N, Li G S, Chin W S, Ji W (2010). J Phys Chem C.

